# Neurobiological and Clinical Markers for a Severe Form of Alcoholism in Women

**Published:** 1995

**Authors:** Shirley Y. Hill

**Affiliations:** Shirley Y. Hill, Ph.D., is a professor of psychiatry and director of alcoholism and genetics research in the Department of Psychiatry, University of Pittsburgh Medical Center, Pittsburgh, Pennsylvania

**Keywords:** biological markers, AOD dependence, female, risk factors, hereditary factors, environmental factors, evoked potential, adoption study, twin study, family study, disorder classification, behavioral and mental disorder, childhood, high risk group

It is now well established that a person’s risk for developing alcoholism^1^ tends to reoccur in families (Hill et al. 1977; Cotton 1979; Hill et al. 1987; Hill 1994). Although alcoholism is clearly familial, the role of genetics in a person’s vulnerability to develop alcoholism continues to be debated. Most researchers would support the notion that some genetic influence occurs. The question to be asked is: How much of the variation between people in susceptibility to alcoholism is explained by genetic factors? It is also important to ask: How is genetic vulnerability moderated by personal factors (e.g., gender, age, and co-occurring psychiatric disorders) and environmental ones (e.g., cultural milieu and shared familial environment)? This review will discuss the role of genetic factors in the development of alcoholism in women. The data were obtained from the author’s large, high-density-for-alcoholism pedigree study^2^ of women. The article will discuss (1) the most recent studies of event-related potential^3^ differences in women and their offspring and (2) the clinical characteristics of their offspring.

## Evidence for Genetic Mediation in Women

Several types of studies traditionally have been used in attempts to clarify the contribution of genetic influences to alcoholism, including family, adoption, and twin studies. Some of these studies support a genetic role in women’s alcoholism.

### Family Data

The first study to investigate the familial transmission of alcoholism separately for men and women alcoholics was completed by Cloninger and colleagues (1978). Applying a model (of transmission) that included both genetic and environmental factors, Cloninger and colleagues (1978) concluded that the difference in alcoholism prevalence observed between men and women resulted entirely from cultural or nonfamilial environmental factors. Based on the most recent epidemiological data, the ratio of male to female alcoholism is 2:1 in the general population (Kessler et al. 1994). Cloninger and colleagues found that equal numbers of alcoholic relatives were counted among both male and female alcoholics in their early study, suggesting that both genders have an equal likelihood that their disorder is inherited or is genetically mediated.

### Adoption Studies

In further support of alcoholism’s familial nature, one line of research has indicated that the disease is more likely to be transmitted within families than are most psychiatric disorders (Merikangas et al. 1985). This high transmissibility could, however, result from the family environment (e.g., exposure to an alcoholic parent) rather than from genetic factors. In seeking to separate these two possible influences, a now-classic study of adopted children that concerned the etiology of alcoholism in men (Goodwin et al. 1973) suggested that transmission can occur even in the absence of exposure to an alcoholic parent. Few adoption studies have been conducted that address genetic factors in women’s alcoholism. Thus, the conclusions that can be reached about genetic transmission based on adoption studies in women are tentative (Hill and Smith 1991; Hill 1993).

Three adoption studies have been conducted that report results for female adoptees, one each in Sweden (Bohman et al. 1981), Denmark (Goodwin et al. 1977), and the United States (Cadoret et al. 1985). The Swedish and Danish studies found too few alcoholic women to warrant any conclusions. However, Cadoret and colleagues (1985) had a sufficiently large sample to conclude that genetic factors operated in the etiology of female alcoholism (for further discussion of adoption research, see the article by Cadoret, pp. 195–200).

### Twin Studies

Further evidence for the genetic mediation of alcoholism in women can be gained from studies that have examined whether members of adult twin pairs match their twins in alcohol-related behaviors. This research compares the agreement in behavior between members of identical twin pairs (i.e., monozygotic [MZ] twins, whose genes are identical), with that of fraternal twin pairs (i.e., dizygotic [DZ] twins, who share an average of 50 percent of their genes). If a study finds twice the rate of agreement in the MZ twins as in the DZ twins, genetic influences are suggested to be at work (for further discussion, see the article by Prescott and Kendler, pp. 200–205). Agreement between members of twin pairs for both drinking behaviors (Heath et al. 1989) and alcohol dependence (Gurling et al. 1981; Pickens and Svikis 1988; Pickens et al. 1991; Kendler et al. 1992; McGue et al. 1992) have been studied.

Although three studies utilizing clinical samples (Gurling et al. 1981; Pickens and Svikis 1988; McGue et al. 1992) did not find that MZ twins differed from DZ twins in a pattern suggestive of a genetic role, recent reports by Pickens and colleagues (1991) and Kendler and colleagues (1992) are more convincing. For example, Kendler and colleagues, studying a sample of more than 1,000 female MZ and DZ twin pairs from the general population, found substantially higher correlations of alcohol dependence between female MZ twins than between DZ twins; they ascribed more than 50 percent of the variation in the degree of alcoholism risk (i.e., variance) seen in their sample to genetic factors (Kendler et al. 1995).

## Evidence for Two Types of Alcoholism in Women

Twin and adoption studies support a genetic component in women’s alcoholism. It is likely, however, that genetic factors make a greater contribution to some women’s development of the disorder than to other women’s. Genetic risk for developing alcoholism appears to exist on a continuum ranging from virtually no risk to exceptionally high risk; the latter designation results from having a concentration of alcoholics in one’s family (i.e., familial density). For simplicity of argument, however, the liability to develop alcoholism can be thought of as having two basic forms, distinguished by the extent to which each is influenced by genetic factors.

Cloninger and colleagues (1981) described two forms of alcoholism among men. Male type I alcoholics are those whose likelihood of drinking depends much more heavily on the environmental conditions under which they reside than on genetic influence. The type II male alcoholic, however, has been described as having familial alcoholism and as developing alcoholism at a much earlier age (i.e., early onset, often during adolescence) and to a more severe degree than the type I male.

Accordingly, it is possible that two types of alcoholism exist in women. For example, studies by Glenn and Nixon (1991) and Lex and colleagues (1991) provide evidence for more severe symptoms in women with early onset alcoholism (i.e., beginning before age 25). Women with the severe form of alcoholism also come from families with a greater density of alcoholism among their members. Likewise, preliminary results from Hill and colleagues suggest a very early onset of alcoholism for female alcoholics whose families display multigenerational alcoholism (discussed below). This profile suggests that a form similar to type II male alcoholism may exist in women.

Some researchers, however, believe that all alcoholism in women is less severe than it is in men. For example, Gilligan and colleagues (1987) have used the term “female-like” to describe a less severe and less genetically mediated form of alcoholism in men (i.e., type I alcoholism). This result was based on an analysis of data from the relatives of female and male alcoholics (i.e., the alcoholics were the primary subjects, or probands) in their study of families of alcoholics. Gilligan and colleagues concluded that greater genetic involvement was present within the families of male alcoholics than within the families of female alcoholics. Moreover, they concluded that two types of alcoholism existed in males, but only one type occurred in females. The researchers did not, however, test for more than one type of inheritance to explain the transmission of alcoholism among the female proband families. Thus, assumptions about the existence of only a single form of alcoholism in women may have been premature (Aston and Hill 1990*a*).

The alcoholism field appears to have accepted the view of two types of male alcoholism while assuming, with a few notable exceptions (Glenn and Nixon 1991; Hill and Smith 1991; Lex et al. 1991), that only one type of alcoholism—one without a genetic etiology—exists in females. In fact, as suggested above, the familial form of alcoholism that appears to exist in women resembles the familial form (i.e., type II) seen in men. Evidence of familial transmission is readily apparent for cases in which alcoholism severity is greater. For example, in families having members with early onset-type alcoholism, in which multiple afflicted relatives are present (sometimes in multiple generations), the tendency for alcoholism to run in families is clear. Nevertheless, familial transmission still could be a result of common familial environmental factors rather than genetic ones. Although the data needed to draw firm conclusions about female alcoholism are missing, the literature reviewed above suggests that alcoholism can be genetically mediated in women. Late-onset alcoholism in women may well be influenced more by environmental factors (e.g., divorce or loss of maternal role in middle age [empty-nest syndrome]) than by genetic factors. To lend support to the existing evidence for more than one (i.e., heterogeneous) form of female alcoholism, detailed studies of the genetic aspects of alcoholism in women and in their families have been conducted and are discussed below.

It may be concluded that the etiology of female alcoholism has as much likelihood of being mediated through genetic factors as does men’s (Hill 1993). The evidence for genetic mediation includes two twin studies of female alcoholism in which heritability was found to be significant (Pickens et al. 1991; Kendler et al. 1992) and an adoption study (Cadoret et al. 1985). Moreover, alcoholism appears to be heterogeneous in women, with one form (the early onset type) much more likely to be influenced by genetic factors than the other (late-onset) form (Glenn and Nixon 1991; Lex et al. 1991; Hill and Smith 1991).

## Genetic Mediation of Alcoholism in Women: Potential Risk Markers

The process of becoming alcoholic still may be different for women than for men (Hill 1993). This possibility suggests a need to search for markers of vulnerability for women as well as for men. Finding biological markers of alcoholism risk that are minimally affected by environmental factors (e.g., exposure to an alcoholic parent, sibling, or other family member) could prove useful in understanding the relative contribution of genetic factors to vulnerability for alcoholism. Additionally, these markers could be utilized in prevention efforts by providing a screen for alcoholism susceptibility.

The search for useful markers of “risk” would be facilitated by the identification of a physiological trait in alcoholics that differs from nonalcoholics. Also, finding a marker that discriminates high- from low-risk persons who are not themselves alcoholic is an important step in this process. Ideally, one also would like to find a marker that is under genetic control. Locating neurobiological markers of vulnerability in women and girls is especially useful if susceptibility varies by gender.

### History of Event-Related Potential Research

**Figure 1:**
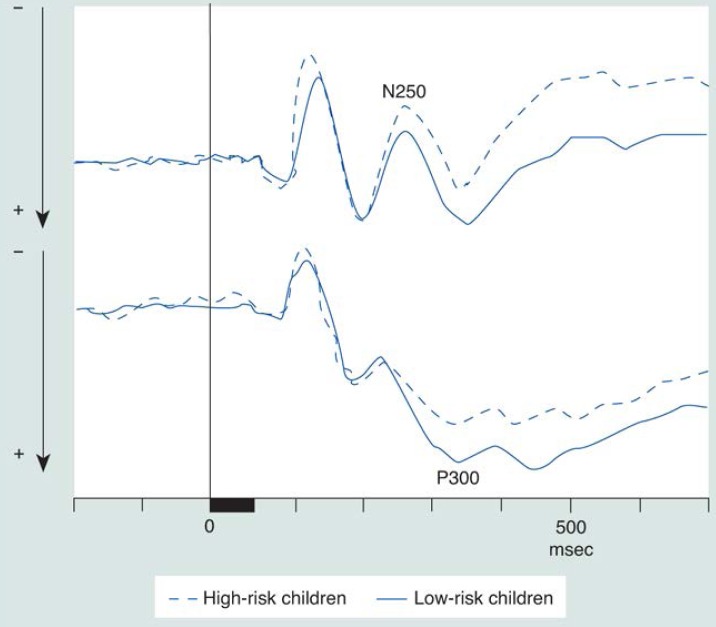
Two forms of electrophysiological evidence show that children at high risk for developing alcoholism (i.e., they have a family history of alcoholism) have decreased brain reactions to stimuli. The two average responses to auditory stimuli that appear on the graphs (i.e., event-related potential wave forms) were recorded from the brains of 35 high- and 35 low-risk children during an auditory task in which the children picked out the “rare” events. In this case, rare high tones were identified as they occurred during a series of low tones.

One biological tool that has proved useful for investigating markers of alcoholism is measurement of the event-related potential (ERP) of the brain. The ERP is measured with the electroencephalogram (EEG), which amplifies the naturally occurring brain electrical activity at the scalp. The ERP is graphically displayed on a scan as a wave with several peaks and valleys (see figure 1). ERP’s occur in response to sensory, motor, or cognitive events. One component, the P300, is one of the positive peaks on the ERP wave and occurs approximately 300 milliseconds (ms) after an informative event. In laboratory tests, the P300 is elicited by asking a subject to respond to an unusual stimulus (e.g., “oddball” paradigms: a high tone in a series of low tones or a red circle in a series of green circles) by performing a simple task (e.g., pressing a button or counting the high tones).

Researchers have examined the P300 both in alcoholics and in nonalcoholic persons at high risk for developing alcoholism. These studies have observed whether the relationship between being an alcoholic or being a high-risk nonalcoholic person is associated with reduced height (i.e., amplitude) of the P300 wave or with the time it takes for the stimulus to be presented and the P300 peak to occur (i.e., the P300 latency). P300 latency changes generally accompany exposure to neuro-toxic substances such as alcohol. Thus, latency changes can be associated with recent exposure to alcohol. ERP studies in male chronic alcoholics have shown evidence for a reduction in P300 amplitude in some investigations (Porjesz and Begleiter 1981), but not in all studies (Pfefferbaum et al. 1979; Hermanutz et al. 1981; Lille et al. 1987; Hill et al. 1988, 1995*b*). Because alcoholics consume alcohol for extensive periods of time and at varying intervals before testing, uncovering P300 differences that may have existed before the onset of abusive drinking is not always possible. Numerous studies, however, have demonstrated deficits in the P300 component in nonalcoholic high-risk children and adolescents. Prior to a report from Hill and colleagues (1995*a*), researchers had found the P300 deficit only in the offspring of male alcoholics (Begleiter et al. 1984; Whipple et al. 1988; Hill et al. 1990; Hill and Steinhauer 1993*a;* Steinhauer and Hill 1993).

The ERP, and the P300 component in particular, has value as a marker of alcoholism for two reasons. First, ERP’s are associated with particular sensory and cognitive aspects of information processing. These processes may be affected in people at risk for alcoholism. Second, the ERP wave form has been found to be more similar between family members than between unrelated people. Thus, it appears to be under genetic control (Steinhauer et al. 1986; Polich and Burns 1987; Aston and Hill 1990*b*) and is unlikely to be influenced by environmental factors. Someone whose P300 amplitude is reduced and who is from a family with a high density of alcoholism could have an inherited risk of developing the disease. Thus, reduced P300 in women at high risk for developing alcoholism could lend support to the idea that the disease can be genetically mediated in women.

Two studies have examined the P300 component in alcoholic women (Parson et al. 1990; Hill and Steinhauer 1993*b*). One of these (Hill and Steinhauer 1993*b*) looked at high-risk relatives of alcoholic women for possible P300 alterations, finding P300 amplitude deficits among the alcoholic women. Parsons and colleagues (1990) compared ERP characteristics of female alcoholics with those of female nonalcoholic control subjects using auditory and visual paradigms but found no differences in P300 amplitude or latency.

## The Biological Factors Family Study of Female Alcoholism

Considering the evidence both questioning and/or supporting the existence of a severe form of genetically influenced alcoholism in women, and given the usefulness of the P300 as a potential marker for genetic vulnerability, Hill and colleagues designed a study to examine the nature of female familial alcoholism. They investigated the presence of a severe form of alcoholism and its relationship to any effects on P300 waves among women and children from families in which a high concentration of female alcoholics was present.

### Criteria for Families

**Figure 2:**
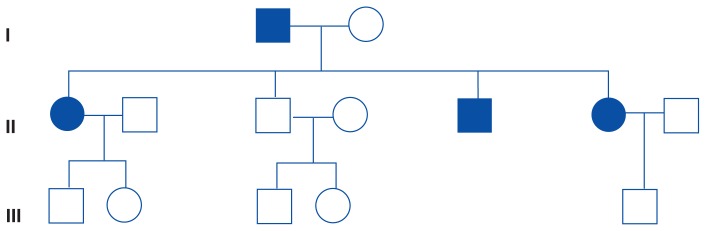
A representative pedigree from the Biological Risk Factors Family Study of alcoholic women and their relatives. The solid squares and circles indicate family members with alcoholism. Squares represent men, and circles represent women. Roman numerals indicate generations. The pedigree represents a family with a high frequency of female alcoholism.

In the study, multiple extended families with multigenerational alcoholism were located over a 6-year period as part of the Biological Risk Factors Family Study (figure 2). One goal of this research has been to identify neurobiological indicators of risk, such as the P300, in alcoholic women and their relatives who are at high risk of developing the disease, particularly minor children from these families.

In contrast to the methodology used by Parsons and colleagues (1990) (mentioned earlier; wherein the FHP designation could refer to just one alcoholic relative being present in a woman’s family) Hill and colleagues’ study ascertained FHP histories with the intention of finding families (i.e., pedigrees) with a high density of alcoholic members (Aston and Hill 1990*a*; Yuan et al. in press). The high-risk families in the study were found initially through a pair of female alcoholic siblings. This strategy results in the greatest chances of alcoholism occurring across generations within the same family. At least one member of each pair was in treatment for alcoholism when identified; if an alcoholic sibling did not exist, the presence of other alcoholic female relatives sometimes enabled the family to be included. All probands and first-degree relatives (i.e., parents, siblings, and children) included in the study were required to be largely free of psychiatric disorders (i.e., recurrent depression and schizophrenia were conditions for exclusion).^4^ This requirement ensured that the neurobiological markers found would be specific to alcoholism and not to general “psychiatric morbidity.” In addition, possible sources of variation were evaluated in all study participants, including socioeconomic status, familial environment, and menstrual cycle phase, along with overall neuropsychological test performance, to ensure that any differences between the groups did not result from these variables.

All first-degree, adult relatives (both alcoholic and nonalcoholic) of the treated alcoholic women were studied. The children studied were the offspring of the alcoholic subjects and their siblings. To arrive at a diagnosis with respect to alcoholism and other psychopathology, two interviews were conducted, or two family history reports were gathered for each relative. The age of onset of alcoholism for women in this study was quite early, with a median of 16 years (Hill 1993).^5^ Because the study families were chosen for their high frequency of alcoholism (a minimum of two alcoholic members per family), the researchers hypothesized that the families’ alcoholism would be genetically mediated (as was intended by research design). The study of high-density families would maximize the likelihood of finding biological markers, should they exist.

The control families (i.e., the low-risk families) were selected for minimal psychopathology (including alcoholism) among the sister pairs and their first-degree relatives. These families were selected so that general psychiatric disorders in the high- and low-risk groups were equivalent (for further review of the methods involved in this study, see Hill 1993; Hill and Steinhauer 1993*a;* Steinhauer and Hill 1993).

## Event-Related Potentials

To verify their hypothesis that the alcoholic women did have a genetically influenced form of the disease, the researchers observed ERP’s in the women and in their children. Adult women and minor children from the high- and low-risk pedigrees were evaluated using identical tests and recording conditions (one visual task and two listening tasks), enabling the assessment of important developmental and gender differences in P300 waves.

### ERP Differences in Alcoholic Women, Nonalcoholic Sisters, and Controls

One phase of the study investigated possible differences in P300 that might be associated with alcoholism risk (Hill and Steinhauer 1993*b*). The subjects studied included 25 alcoholic women, 31 of their high-risk nonalcoholic sisters, and 30 control women from low-risk families.

#### Amplitude Is Reduced

The P300 amplitude elicited from one auditory task was reduced significantly for the alcoholic women compared with the control women and their non-alcoholic sisters. For the other auditory and the visual task, alcoholic women also were found to have reduced P300 amplitude compared with their nonalcoholic sisters. P300 latency did not differ by group. This suggests that group differences in P300 did not result from differences in recent exposure to alcohol. The findings from this portion of the study suggest that reduction in P300 amplitude occurs only in alcoholic women.

#### Significance of Findings

The difference between the P300 amplitude of alcoholic women and that of their nonalcoholic sisters is of considerable significance. Although the reduction could result from the longstanding use of alcohol leading to neuropathological changes, this possibility appears unlikely because the women showed equal ability on tests of neuropsychological functioning. Any genetic influence on women in these families may be inherited by one sister and not by the other. In addition, a recent analysis of a large data set from alcoholic men, their high-risk brothers, and nonalcoholic controls showed no P300 amplitude reduction in adult males (Hill et al. 1995*b*), a result also found by others (Pfefferbaum et al. 1979; Hermanutz et al. 1981; Lille et al. 1987). These results have led to speculation that P300 differences in high-risk populations may represent delays in development of cognitive functioning that typically are seen only in childhood. In contrast to findings in men, women who became alcoholic in adolescence persist in having reduced P300 amplitude in adulthood. Further research is needed to examine the possible gender differences (e.g., differences in the hormonal system) that might account for this phenomenon. At any rate, women alcoholics display clearly different neurophysiological characteristics from their nonalcoholic sisters, a condition that may have its antecedents in childhood. Demonstration of differences in childhood, prior to the initiation of drinking, would provide further evidence that the marker has etiological significance and is not merely a consequence of drinking.

### P300 in High-Risk Children From Female Alcoholism Families

Because alcoholic women exhibit P300 amplitude reduction, their children also might have reduced P300, possibly indicating a genetic risk for developing alcoholism.

#### Subjects

Seventy-six children ages 8 to 18 were studied. Two groups of children (whose mean age was 11.3) were drawn from the high-risk families and the control families. Both groups contained 38 children: 17 males and 21 females. All children were free of alcohol and other drug (AOD) use at the time of testing, and only one child was a regular AOD user.^6^

Because the mothers of both groups of children could have consumed alcohol during pregnancy, the study design included interviewing all mothers (even those who were social drinkers) about current and life-time AOD use to determine AOD use during pregnancy. Mothers of 12 high-risk children reported drinking during pregnancy (2 of the mothers also had used marijuana), and mothers of 26 children denied AOD use during pregnancy.

#### Results for an Auditory ERP Task

The P300 peak was studied in 35 high-risk children and 35 control children (matched in age). A significant effect was found in one task, in which the amplitude for the high-risk group was lower than that for the low-risk group (Hill et al. 1995*a*). To control for prenatal alcohol exposure, data were reanalyzed to exclude the high-risk children whose mothers drank during pregnancy and their corresponding controls. Group differences remained even in this smaller sample of 24 children of mothers who reported abstinence when compared with control subjects. Thus, the P300 amplitude reduction in high-risk children appears to result from a familial vulnerability and not from prenatal alcohol exposure.

#### Reduced P300 Amplitude in Girls From Female Alcoholism Families

**Figure 3:**
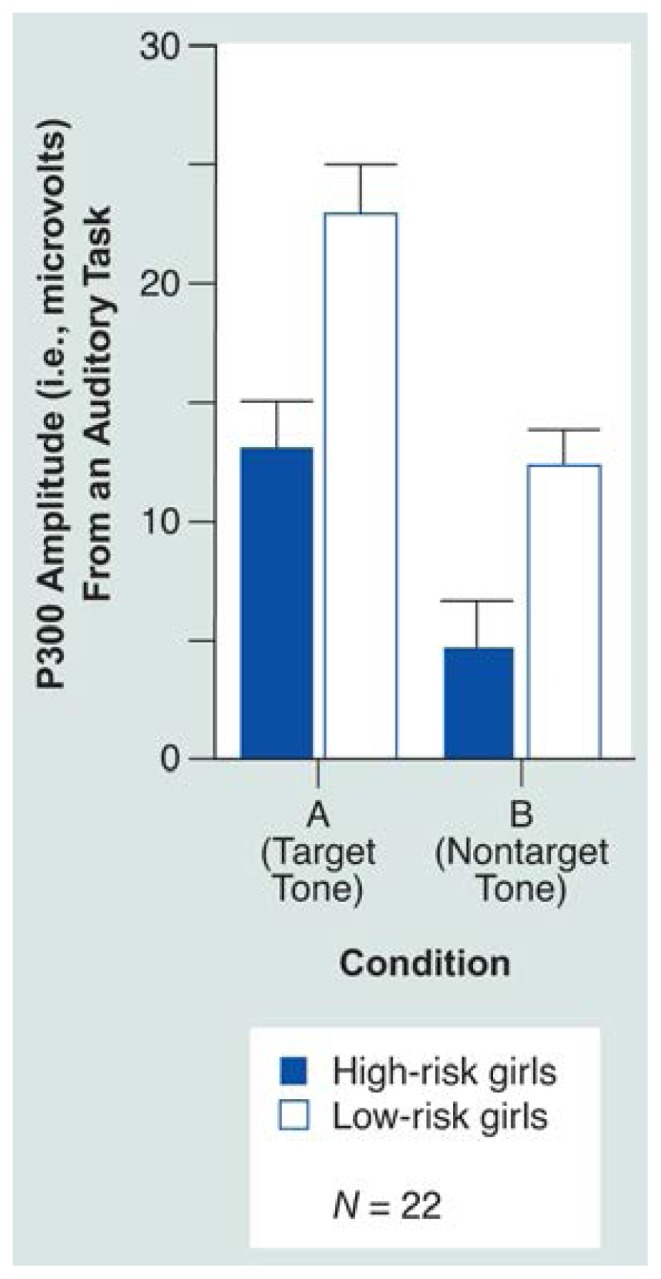
P300 response to an auditory task in 11 daughters of alcoholic women (i.e., high-risk girls) and 11 age-matched control girls. Two conditions are shown: the target tone (i.e., the “rare” tone that subjects are asked to acknowledge) (A) and the nontarget tone (i.e., one of the tones the subjects hear but do not acknowledge) (B). As shown, P300 response is lower among high-risk girls.

Because the study also was intended to discern whether female children would exhibit ERP characteristics similar to those found previously among high-risk male children, it focused on reanalyzing data with respect to P300 differences in daughters of alcoholic mothers (figure 3). Because a reduction in P300 amplitude might result from the joint influence of having a biological mother and father who both were alcoholic, for this analysis, girls who had an alcoholic father were excluded. Female children with alcoholic mothers did display reduced P300 amplitude (Hill et al. 1995*a*).

The P300 results from the girls with alcoholic mothers suggest that the same neurobiological indicators of risk found in children from high-density-for-male-alcoholism families (Hill and Steinhauer 1993*a*; Steinhauer and Hill 1993) are risk indicators in the high-density-for-female-alcoholism families. Interestingly, compared with low-risk boys, a greater proportion of high-risk boys exhibit P300 amplitude below the median of the control subjects (approximately one in three). A similar comparison for girls showed that approximately one in six high-risk girls exhibited P300 below the median of the controls. Thus, because alcoholism among females is less prevalent in the general population than it is among males, one would expect the ratio of girls to boys showing P300 reduction to be comparable to the ratio of female to male alcoholics in the general population. Although this study of offspring of alcoholic women has not yet followed the children prospectively, P300 appears to predict clinical outcome for alcohol dependence. This hypothesis is based on results from a companion study of children from male alcoholic families (Hill et al. 1995*c*). In this 8-year followup study of children from high-risk-for-male alcoholism families, a relationship between P300 amplitude at age 10 and alcohol dependence existed at followup. Although it remains to be determined whether the childhood P300 reduction in the high-risk-for-female alcoholism families is specific to alcoholism or is a general marker of adult psychopathology, the repeated observations of differences between high- and low-risk children warrant long-term followup of these children.

## Psychopathology in Children From Female Alcoholism Families

The importance of studying psychopathology in offspring from maternal alcoholism families is twofold. Psychopathology in children increases their risk for adult disorders, including alcohol dependence. Also, developing appropriate interventions for these children requires identifying both the type and extent of these problems.

Hill and colleagues evaluated all available children in their sample who were between ages 8 and 18 for risk of psychopathology.^7^ The high-risk children did not have significantly higher rates of particular childhood disorders (e.g., affective disorders, anxiety disorders, oppositional/ conduct disorders, or attention deficit disorders). When the total number of diagnoses were compared, however, the high-risk children showed significantly higher total rates of psychopathology.

Next, the researchers asked whether parental alcoholism had any specific link to higher rates of psychopathology among these children. They examined the children’s particular psychopathology and its relationship to three major variables: the mother’s and custodial father’s^8^ alcoholism diagnosis and the age group of the child (under age 12 versus age 13 and over)(Hill and Muka in press). Children who had only an alcoholic mother were almost five times as likely to develop psychopathology compared with low-risk children, regardless of their ages. Having, in addition, a custodial father who was alcoholic increased a child’s relative odds of developing psychopathology. Moreover, these results suggest the particular importance of alcoholic role models as risk factors during adolescence; children over age 13 who did not have a custodial father who was alcoholic had a risk of 4.94 compared with a 30-fold increase in risk when both parents were alcoholic.

Because the children evaluated were still, on average, quite young, it remains to be determined how many will succumb to an AOD-use disorder,^9^ particularly alcohol dependence. However, the recurrence of alcoholism observed in more than one generation of the families studied in this research (approximately 50 percent of the relatives of these alcoholic women are alcoholic) suggests that these children have a greatly increased risk for alcoholism by adulthood. Long-term followup of children whose mothers have early onset familial alcoholism will establish the rates of alcohol dependence among offspring of these families and verify the preliminary observations that the neurobiological marker observed in children is a predictor of risk; that is, those with lower P300 will succumb more often to AOD-use disorders.

## Summary and Conclusions

Review of the literature suggests that the existence of a genetically influenced form of alcoholism is as likely for women as it is for men. Similarly, two types of alcoholism appear to occur in women: (1) an early onset type that often develops before age 21 and occurs in women whose families have a particularly high frequency of alcoholism among their members and (2) a late-onset (i.e., midlife) type that appears to be associated with a peak in heavy drinking that occurs at this time (Hill 1993) and that is more likely determined by environmental variables (e.g., loss of spouse). Early onset alcoholism in women represents a severe form of the disorder, with recurrence from generation to generation. Prevention efforts may be especially important for children from these families.

Correlated with the presence of this severe form of alcoholism in families is a reduced P300 wave among some family members. Previously, when data were compared for children from families with a high density of male alcoholism, a number of striking differences in ERP characteristics emerged, including reduced P300 in high-risk children compared with control subjects (Hill et al. 1990; Hill and Steinhauer 1993*a*; Steinhauer and Hill 1993). Recent research has shown that children of female alcoholics display similar P300 amplitude reduction (Hill et al. 1995*a*). This finding suggests that P300 is a neurobiological marker for genetic alcoholism risk in children from both male and female alcoholism families.

Comparison of the clinical and psychopathological characteristics of children from high-risk pedigrees, in which approximately 50 percent of both male and female relatives are alcoholic, reveals that these children are at greatly increased risk for childhood psychiatric disorders relative to controls. This conclusion stands in contrast with the more modest differences in psychopathology reported for children from high-risk families located through male alcoholics (Hill and Hruska 1992). Thus, it appears that the consequences of being a child of an alcoholic woman are represented by significant neurobiological markers of risk (e.g., reduction in P300 amplitude) and psychopathological conditions that may promote alcoholism.

## Footnotes

1 As it is used in this article, the terms “alcoholism” and “alcohol dependence” refer to a disease characterized by abnormal alcohol-seeking behavior that leads to impaired control over drinking.

2 The term “high density for alcoholism” refers to families in which the chance of a relative of the alcoholic’s being alcoholic is far greater than it is in the general popultion (in Hill and colleagues’ families, their risk is five times higher than in the general population).

3 The event-related potential is a specific measure of the brain’s electrophysiological response to a perceived stimulus.

4 Psychiatric disorders were defined by the American Psychiatric Association’s *Diagnostic and Statistical Manual of Mental Disorders, Third Edition*.

5 In the general population, one-half of all alcoholic women experience onset after the age of 45, whereas for men, one-half are alcoholic by age 30 (Helzer et al. 1991).

6 For clarity, the term “alcohol and other drug (AOD) use” was used to standardize terminology throughout this article. However, the author used criteria listed in the American Psychatric Association’s *Diagnostic and Statistical Manual of Mental Disorders, Third Edition*, which refers to “alcohol and/or drug use disorders.”

7 Lifetime rates of childhood psychopathology, as determined from the Schedule for Affective Disorders and Schizophrenia for School-Aged Children (K-SADS) (Orvaschel et al. 1982; Chambers et al. 1985), were compared for the two groups of children (i.e., the 38 at high risk for alcoholism and 38 control subjects at low risk).

8 The custodial father is defined as a biological, step-, or adoptive father with whom the child resided at the time of evaluation. A total of 20 of the 38 custodial fathers were reported to be the biological father.

9 The followup study will investigate AOD-use disorders as they are defined in the American Psychiatric Association’s *Diagnostic and Statistical Manual of Mental Disorders, Fourth Edition*.
